# A Breast Cancer Candidate Locus at 6q Narrowed to 6q15-q21

**DOI:** 10.3390/genes15020218

**Published:** 2024-02-08

**Authors:** Dorottya Csuka, Edda S. Freysteinsdottir, Gudrun Johannesdottir, Bjarni A. Agnarsson, Oskar Th. Johannsson, Rosa B. Barkardottir, Adalgeir Arason

**Affiliations:** 1 Molecular Pathology Unit, Department of Pathology, Landspitali—The National University Hospital of Iceland, 101 Reykjavik, Iceland; dorottyab@landspitali.is (D.C.); rosa@landspitali.is (R.B.B.); 2 Department of Pathology, Landspitali—The National University Hospital of Iceland, 101 Reykjavik, Iceland; 3 Department of Medical Oncology, Landspitali—The National University Hospital of Iceland, 101 Reykjavik, Iceland; 4 BMC (Biomedical Center), Faculty of Medicine, University of Iceland, 101 Reykjavik, Iceland

**Keywords:** hereditary breast cancer, non-BRCA1/2, oligogenic cancer susceptibility

## Abstract

Although a number of high-risk breast cancer genes have been identified, including *BRCA1* and *BRCA2*, the risk profile of many high-risk families cannot be explained using known breast cancer genes. Previously, we have shown strong indications of new breast cancer risk loci at chromosomes 2p, 6q, and 14q in a family of six generations including 10 breast cancer cases. In this study, we identified and traced four new family branches descending from siblings of the parents in the top generation of the studied family. One distantly related branch included four breast cancer cases, two of whom were diagnosed at age < 45 years. DNA samples from the cases were typed at selected polymorphic markers from all three chromosome loci, to test identical origin of the haplotypes. All four cases were shown to segregate a common 6q haplotype with a region identical to the previously identified 6q haplotype. The data strongly support a new breast cancer locus at 6q, and narrow it down to a 17 MB interval at 6q15-q21.

## 1. Introduction

With respect to clinical family history, most breast cancer (BC) cases are sporadic, but up to 20% are familial, with at least one additional case among first or second degree relatives. Approximately 5–10% of BCs are considered hereditary (HBC) (reviewed in [1]), either by the degree and pattern of familial aggregation of cases or by positive testing of mutations in known genes (or both). Alleles contributing to heredity differ in risk magnitude. While high- and moderate-risk alleles are rare, those conferring low risk have a much wider range of allele frequencies and are hard to detect, especially if they are infrequent [2]. Furthermore, known high- and moderate-risk alleles are confined to a low number of genes, whereas low-risk alleles occur at multiple (at least 150) widely distributed chromosomal loci [3]. Low-risk alleles can collectively cause substantial BC risk in an individual or families, but the major genetic explanation for multiple-case HBC families lies, thus far, in single high-risk genes. Most such families are explained by a mutation in either of the well-known breast-ovarian cancer genes *BRCA1* and *BRCA2*, which account for approximately 10–15% of cases with familial risk of breast cancer. Although mutations in some other high-risk genes (*TP53*, *STK11*, *CDH1*, *PTEN*) and moderate-risk genes (*ATM*, *CHEK2*, *PALB2*) are also known to increase BC risk, a clear genetic explanation is still missing in over 50% of familial cases [1].

In a genome-wide search of new chromosomal loci in high-risk BC [4], we earlier reported a pedigree with cosegregation of three haplotypes, located at chromosomes 2p, 6q, and 14q, in nine breast cancer-affected relatives, thus indicating an oligogenic cause of HBC in this family. All three loci had LOD (logarithm of odds) scores close to statistical significance, which is rarely seen for a locus on a single family basis. In some other studies, notable linkage signals had been seen at two or three chromosome regions in the same family [5,6,7,8]. In our report, we genotyped our indicated loci in a collection of Nordic non-*BRCA1/BRCA2* families and found support for the risk indication of the 6q locus. The region of this locus does not overlap with the position of the *ESR1* gene at 6q25.1-q25.2 or with the more recently suggested breast cancer susceptibility locus on 6q14.1 [9], and it has not been reported in other studies as a BC risk locus. Whether this locus acts alone to confer its risk, or has its risk elevated in the genetic background of other loci, including 2p and 14q, is not known. It also remains to be confirmed whether 2p, 6q, and 14q harbor BC risk genes, and if they do, there is a need to identify them. None of these three loci showed, per analysis of their wt-LOH (wild-type loss of heterogeneity), any indications of harboring a tumor suppressor gene [4].

The methodology of identifying disease-causing genes has changed profoundly in the last 30 years, given the current facility of whole-exome or even whole-genome next-generation sequencing (NGS) to identify genes with pathogenic mutations. This is a stark comparison to the days of the discovery of the first HBC genes, *BRCA1* [10] and *BRCA2* [11], when positional cloning was necessary. Such work depended on linkage analysis and identification of chromosome recombination in pedigrees, in order to pinpoint the exact chromosomal location of the gene in question, before it could be sequenced in search of mutations. In the case of our above-mentioned pedigree, the causative genes will not be easily identified without the support of positional cloning because of a lack of clearly pathogenic mutations in genes in the three regions, discovered during our attempts to use whole-exomic NGS. The aim of identifying the causal genetic variants in question therefore motivates a refining and narrowing of the chromosomal search regions. In our current study, we expanded the study of the oligogenic pedigree by tracing additional family branches related to the parents in the top generation and looked for signs of cosegregation of the indicated loci with BC. We now report further support for the 6q locus and refine its search region by added recombinations.

## 2. Materials and Methods

**Figure 1:**
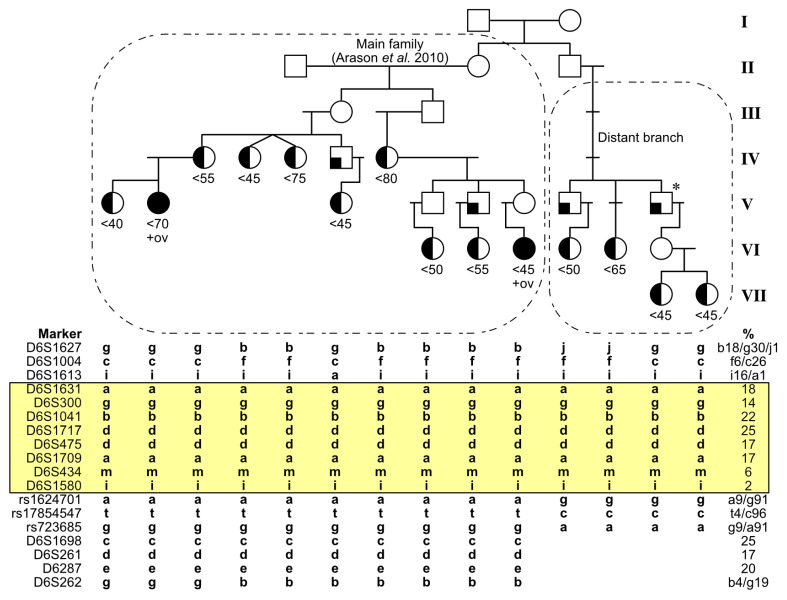
Extended pedigree tracing showing the narrowed candidate region at chromosome 6q. Not all unaffected family members are shown, and the pedigree is somewhat distorted in order to avoid recognition. The upper panel shows the extended pedigree of family 70234 [4], where half-filled symbols denote diagnosis of BC (with approximate ages shown under the symbols) and full black filling denotes also ovarian cancer (ov) as well as BC. Symbols with a filled corner indicate diagnosis of prostatic or other cancers. Circles refer to females and boxes to males. Asterisk refers to the grandfather of two sisters, who had a haplotype identical to the remaining two cases (his nieces) in the new branch. The breast-ovarian cancer case in generation VI is also a *BRCA2*-mutation carrier and therefore not included in the count of 6q-haplotype carriers among BC cases in this report. The lower panel shows haplotype-contained alleles of analyzed markers, together with their allele frequencies in Icelandic controls. Alleles are coded with a, c, g, or t in case of rs-markers, and with these or other lower case letters in case of microsatellite markers (after selecting the codes for each marker by pairing alphabetical order with increasing population allele sizes). Alleles that are identical in all genotyped affected family members are highlighted in yellow.

Our study is based on an extensively traced seven-generation Icelandic pedigree, where detailed clinical and laboratory data are available from the following affected family members: Ten subjects in the main family branch diagnosed with BC (two of them diagnosed with ovarian cancer as well, but one of these was also a carrier of a *BRCA2* mutation and is therefore omitted in the count of 6q-haplotype carriers) [4], and a further four cases with BC in a distantly related family branch (Figure 1). Pedigree and clinical data were obtained from the Genetic Committee of the University of Iceland and the Icelandic Cancer Registry. For estimation of approximate population allele frequencies, 59 blood samples collected at random from the adult Icelandic population were also genotyped.

Written informed consent was obtained from all study subjects and approval of the National Bioethics Committee was also obtained (reference number VSN-11-105-V9). The study was conducted in accordance with the Declaration of Helsinki.

DNA extraction was performed from whole blood using a salting-out procedure and from frozen normal tissue samples with the Wizard Genomic DNA Purification kit (Promega, Madison, WI, USA). Screening for the *ATG5* rs1624701, *SLC22A16* rs723685, and *SEC63* rs17854547 polymorphisms was carried out via PCR amplification of selected regions followed by DNA sequencing. Microsatellite markers were amplified with fluorescently labelled PCR primers, followed by fragment analysis. For both the DNA sequencing and the fragment analyses, the PCR products were run on an ABI PRISM 3130 × l Genetic Analyzer automated sequencer (Applied Biosystems) and then analyzed accordingly with either the Sequencher 5.0 (Gene Codes Corporation, Ann Arbor, MI, USA) or the GeneMapper software v4.0 (Applied Biosystems, Foster City, CA, USA). Primer sequences and PCR conditions are available upon request. Detailed methods have been published previously [4].

## 3. Results

In the absence of knowledge of where the 2p, 6q, and 14q haplotypes entered the published pedigree, we sought to extend pedigree tracing to both parental sides in the top generation. The father and mother in that generation each had two siblings. We traced their descendants and assessed these four new pedigree branches for clinical history of cancer, with focus on familial clusters of BC. On the father’s side, one branch had two sub-branches with BCs diagnosed at age < 60. There were three such cases in each sub-branch. None of these cases had signs of having inherited haplotypes related to the previously reported 2p, 6q, or 14q haplotypes. On the mother’s side, one branch had four cases of BC, two of whom were diagnosed at age < 45 years (Figure 1, generation VII). All four turned out to segregate a common 6q-haplotype with a region of alleles identical to the published haplotype and had tested *BRCA1/BRCA2* mutation-negative. The region was typed with additional markers to test these indications of common origin, and to fine-map the region boundaries. As shown in Figure 1, eight markers define a haplotype common to the one in the published pedigree, containing markers with a linked-variant allele frequency down to ~2% in Icelandic control samples.

For the purpose of defining the current search region of the indicated breast cancer risk factor at chromosome 6q, a recombination analysis is important. Above the common region shown in Figure 1 (yellow), genetic recombinations were observed both in the published pedigree and in the new branch. Two occurred between D6S1004 and D6S1613 and one above D6S1004 (left- and right-most cases in Figure 1). One case in the published pedigree revealed a previously undetected recombination below D6S1613; as this was an early-onset case (age < 45 at diagnosis), this supports a further narrowing down of the upper boundary. Below the common region, three markers were typed in all samples, and all indicate a recombination below D6S1580 (Figure 1).

The boundaries of the common haplotype therefore lie between the markers D6S1613 at 6q15 and rs1624701 at 6q21. These correspond to a region of length less than 17 MB (chromosome-6 positions 89.8 to 106.3 MB according to human assembly GRCh38/hg38), which compared to the previously published region of 47.7 MB is a substantial reduction of the search region containing the proposed HBC locus.

## 4. Discussion

We have found supportive evidence for a HBC locus at chromosome 6q and narrowed its candidate region down to a 17 MB interval between D6S1613 and rs1624701. This region contains 61 known genes.

We also looked for the appearance of alleles from the 2p and 14q haplotypes in breast cancer cases in the new distant pedigree branches. There were no indications of these haplotypes, so we could not see where they had entered the main pedigree. We were aware of this possibility in our research plan, since in our previous work extending such large (>5 generations) *BRCA1* or *BRCA2* pedigrees in similar ways as here, we have not observed the relevant mutations in the new distant branches (unpublished results). The probability of each sibling of the original parental generation having inherited the mutation is 50%, and then it attenuates down the generations, since every homozygous non-*BRCA1/BRCA2* genotype has 0% probability of segregating the mutation to children. To add further difficulty, cancer registries provide much less evidence of long past diagnoses in the upper generations, making it more difficult to trace whether segregation of cancer risk to lower generations accords with an origin in the top of the pedigree. In our experience, when we have seen indications of HBC in the lower generations of such distant branches, they were not explained by the main pedigree’s mutation. The absence of 2p- and 14q-haplotypes in the new distant branches here does not make it less likely than before that the strong cosegregation in cases within the main pedigree is a true reflection of HBC risk factors being segregated with these loci. On the other hand, the difficulty of finding new carriers in distant relatives by extending the tracing of such a large pedigree, like we do here, underscores the importance of finding the 6q haplotype being segregated to the four new cases in this study.

When comparing our findings to other published work trying to map new HBC loci, the 2p and 14q loci have an overlap with regions indicated by large families in previous GWS studies of breast cancer [5,7]. The 6q-locus at 6q15-q21 has only been reported by us in this and our previous publication [4]. In 13 Nordic families, supportive indications for this chromosome 6q locus were seen (heterogeneity logarithm of the odds (HLODs) ranging from 0.34 to 1.37 by country subset) [4]. Our present findings now add an important support for this locus.

A study of a candidate breast cancer susceptibility locus on 6q14.1, proximal to our 6q-region, described unsuccessful attempts to find a causative gene by exomic sequencing, because no pathogenic mutations were found in any candidate gene of the region [9]. We have similarly performed a whole-exome sequencing of three carriers of the 2p-, 6q-, and 14q-haplotypes without seeing a mutation indicative of gene knockout or of pathogenicity (unpublished results). Therefore, the genes in question may be more subtly affected, even by mutations in gene control regions outside of exons. The proposed gene at 6q15-q21 could possibly exist with other hard-to-find mutations in other HBC families. Genome-wide association studies of SNP markers in search of BC risk modifier loci (polygenic model) may have missed this association if the gene in question has infrequent mutations that arise in different versions of its haploblock. Further work needs to incorporate whole-genome sequencing of haplotype carriers and possibly the addition of unrelated pedigrees in order to pinpoint mutations that may be subtle, but at least present in the same gene in different families.

## 5. Conclusions

Our study supports the mapping of a HBC locus to chromosome 6q15-q21, emphasizing the need to define the gene in question and its cancer risks, which could aid clinical genetic counseling. Putative risk alleles may be characterized by subtle effects rather than by knocking out the functions of the gene.

## Data Availability

Primer sequences and PCR conditions are available upon request.
